# Intersectional Strategies for Targeting Amacrine and Ganglion Cell Types in the Mouse Retina

**DOI:** 10.3389/fncir.2018.00066

**Published:** 2018-08-22

**Authors:** Andrew Jo, Jian Xu, Sercan Deniz, Suraj Cherian, Steven H. DeVries, Yongling Zhu

**Affiliations:** ^1^Department of Ophthalmology, Feinberg School of Medicine, Northwestern University, Chicago, IL, United States; ^2^Department of Physiology, Feinberg School of Medicine, Northwestern University, Chicago, IL, United States

**Keywords:** retina, Cre, Flp, intersection, amacrine cell, ganglion cell

## Abstract

The mammalian retina harbors over 100 different cell types. To understand how retinal circuits work, it is essential to systematically access each type. A widely used approach for achieving targeted transgene expression exploits promoter-driven Cre lines. However, Cre expression in a given transgenic line in the retina and elsewhere in the brain is rarely confined to a single cell type, contributing ambiguity to the interpretation of results from broadly applied manipulations. To obtain unambiguous information about retinal processing, it is desirable to have strategies for further restricting transgene expression to a few or even to a single cell type. We employed an intersectional strategy based on a Cre/Flp double recombinase system to target amacrine and ganglion cell types in the inner retina. We analyzed expression patterns in seven Flp drivers and then created combinational mouse lines by selective cross breeding with Cre drivers. Breeding with Flp drivers can routinely remove labeling from more than 90% of the cells in Cre drivers, leading to only a handful cell types, typically 2–3, remaining in the intersection. Cre/Flp combinatorial mouse lines enabled us to identify and anatomically characterize retinal cell types with greater ease and demonstrated the feasibility of intersectional strategies in retinal research. In addition to the retina, we examined Flp expression in the lateral geniculate nucleus and superior colliculus. Our results establish a foundation for future application of intersectional strategies in the retina and retino-recipient regions.

## Introduction

The retina is an experimentally tractable preparation for understanding how nervous systems abstract complex information from the environment, including information about object motion, orientation, and permanence under different light conditions (Wassle, 2004; Masland, 2012; Demb and Singer, 2015). Retinal neurons comprise three major classes: the primary sensory cells which include rods and cones; interneurons which include horizontal, bipolar and amacrine cells (ACs); and, the output neurons, retinal ganglion cells (RGCs), that project to retino-recipient areas in the brain.

A diversity of cell types in the retina provides the infrastructure necessary for visual signal processing. There are at least 40 types of RGCs, 50 types of ACs, 14 types of bipolar cells, 2 types of horizontal cells, and 3 types of glial cells (Masland, 2012; Demb and Singer, 2015). Each of the RGC types is tuned to respond best to different and sometimes complex features in a visual scene (Masland, 2012; Sanes and Masland, 2015; Baden et al., 2016). The unique response properties of each RGC type originates from the interplay between excitation and inhibition centered around the RGC dendrites. An RGC type receives excitatory inputs from a subset of the cone bipolar cell types and inhibitory inputs from a subset of AC types (Masland, 2012; Demb and Singer, 2015). Due to the large number of cell types involved and their extensive process ramifications in the innerplexiform layer (IPL), a connectivity diagram containing both functional and anatomical information for the bipolar and AC circuits that feed into each ganglion cell type is unresolved.

Physiological, molecular, and genetic approaches have been used to gain access to specific amacrine and ganglion cell types in order to systematically study their organization and function. One of the most prominent approaches involves the use of genetically modified mouse lines that heritably express transgenes such as GFP or Cre under gene specific promoters (Haverkamp et al., 2009; Siegert et al., 2009; Ivanova et al., 2010). Recently, we analyzed more than 20 Cre drivers that allowed us to distinguish and access more than half of the cell types in the retina (Zhu et al., 2014). While “one-component” transgenic lines (Allen and Luo, 2015) have greatly facilitated the study of retinal function, a persistent problem is that nearly every Cre driver or GFP line targets multiple cell types (Zhu et al., 2014; Martersteck et al., 2017). This multiplicity makes functional studies more difficult by degrading the specificity of broadly applied optogenetic and chemogenetic manipulations. There is a pressing need to further restrict retinal transgene expression to a few or even one neuron type in order to obtain unambiguous information about processing.

**FIGURE 1:**
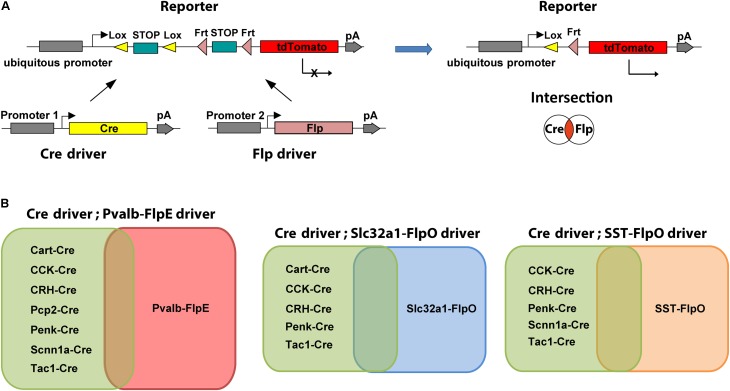
Intersectional strategy with Cre/Flp dual recombinase. **(A)** Schematic diagram of the intersectional strategy with Cre/Flp dual recombinase. The Cre driver line uses gene specific promoter 1, the Flp driver line uses gene specific promoter 2, and the double reporter uses a ubiquitous promoter (left). Expression of tdTomato in the reporter line (*Ai65*) is prevented by lox- and frt-flanked STOP cassettes. Following removal of both STOP cassettes, tdTomato is expressed in the cells that are in the overlap or “intersection” of the two populations of neurons targeted by either the Cre driver or the Flp driver (right). **(B)** Intersectional strategies provide flexibility for targeting specific cell types. Each Flp line can be crossed with many Cre lines to create multiple intersections.

We employed an intersectional strategy in which transgene expression is defined by the combination of two recombinases each driven by a distinct gene promoter (Dymecki et al., 2010; Madisen et al., 2015; He et al., 2016). To date, the most commonly used intersectional combination is the Cre and Flp dual-recombinase system that simultaneously uses both Cre/loxP and Flp/FRT combination to remove a STOP cassette or to reverse a double-floxed inverse open reading frame (DIO/FLEX) (Atasoy et al., 2008; Sohal et al., 2009). Either editing event can lead to the activation of a reporter/effector solely in cells expressing both Cre and Flp recombinases (**Figure 1A**). A prerequisite for the efficient application of an intersectional strategy is to identify the candidate Cre and Flp drivers for crossing. The preferred pair of Cre and Flp drivers should be controlled by different promoters that have overlapping expression patterns in the cells of interest. To facilitate this approach, we have characterized the retinal expression patterns in available Cre and Flp drivers. Based on the individual expression patterns, we crossed lines to produce restricted intersectional expression (**Figure 1B**). A rational extension of this approach can make use of retinal transcriptomic information to design additional Cre and Flp drivers.

The intersectional approach has been recently used in the nervous system to anatomically map and functionally study genetically-defined subpopulations of neurons (Madisen et al., 2015; He et al., 2016), but its effectiveness in retina has not been demonstrated. Here, we screened and analyzed seven new Flp drivers for expression patterns in the retina, lateral geniculate nucleus (LGN), and superior colliculus (SC). We then created and characterized multiple combinational mouse lines by selective cross breeding of Flp and Cre drivers. Our results demonstrate that an intersectional recombination-based system is a powerful tool for increasing the specificity of cell-type targeting in the retina.

## Materials and Methods

### Animals

All the Flp drivers, Cre drivers and reporters were obtained directly from Jackson Laboratory or individual investigators (**Table 1**). Adult mice (5–6 weeks old) of either sex were used for experiments. All animal procedures were performed in accordance with the Guide for the Care and Use of Laboratory Animals as adopted and promulgated by the US National Institutes of Health. All procedures for testing and handling were approved by the Institutional Animal Care and Use Committee of Northwestern University. Cre expression in the *CAGGCreER/Pvalb-FlpE* and *UBC-CreER2/Sst-FlpO* mice relied on “leakiness” without tamoxifen induction. To enhance Cre expression in the *UBC-CreER2*/*Vip-FlpO* mice, single injection of tamoxifen (20 μg, Sigma) was applied intraperitoneally, and retinas were collected after 2–3 days.

**Table 1:** Mouse lines used in this study.

Mouse line	Full name	Source
*Pvalb-FlpE*	B6.Cg-*Pvalb^tm2.1(FLPe)Hze^*/J	JAX 021191
*Sst-FlpO*	*Sst^tm3.1(flpo)Zjh^*/J	JAX 028579
*Vip-FlpO*	*Vip^TM2.1(flpo)Zjh^*/J	JAX 028578
*Slc32a1-FlpO*	B6.Cg-Slc32a1^TM1.1(flpo)Hze^/J	JAX 029591, Hongkui Zeng
*Rorb-FlpO*	B6.Cg**-***Rorb^TM3.1(flpo)Hze^*/J	JAX 029590
*Nkx2-FlpO*	Nkx2-1^tm2.1(flpo)Zjh^/J	JAX 028577
*Dlx5/6-FlpE*	Tg(mI56i-flpe)39Fsh/J	JAX 010815
*CMV-Cre*	B6.C-Tg(CMV-cre)1Cgn/J	JAX 006054
*Vglut2-Cre*	Slc17a6^tm2(cre)Lowl^/J	JAX 016963
*Slc32a1-Cre(VGAT-Cre)*	Slc32a1^tm2(cre)Lowl^/J	JAX 016962
*UBC-CreER2*	B6.Cg-Ndor1^Tg(UBC-cre^**^/^**^ERT2)1Ejb^/1J	JAX 007001
*CAGGCre-ER*	B6.Cg-Tg(CAG-cre/Esr1^∗^)5Amc/J	JAX 004682
*Cck-Cre*	Cck^tm1.1(cre)Zjh^/J	JAX 012706
*Pcp2-cre*	B6.129-Tg(Pcp2-cre)2Mpin/J	JAX 004146
*Crh-Cre*	B6(Cg)-*Crh^TM1(cre)Zjh^*/J	JAX 012704
*Cart-Cre*	B6;129S*-Cartpt^TM1.1(cre)Hze^*/J	JAX 028533
*Penk-Cre*	B6;129S*-Penk^tm2(cre)Hze^*/J	JAX 025112
*Ai9*	B6.Cg-Gt(ROSA)26Sor^tm9(CAG-tdTomato)Hze^/J	JAX 007909
*Ai65*	B6;129S-Gt(ROSA)26Sor^tm65.1(CAG-tdTomato)Hze^/J	JAX 021875

*JAX, the Jackson Laboratory*.

### Immunohistochemistry and Imaging

For immunohistochemistry in the retina, mice were euthanized, and eyes were fixed with 4% paraformaldehyde for 1h and then retinas were dissected. Retinas were washed with a modified phosphate buffer (PB) containing 0.5% Triton X-100 and 0.1% NaN_3_, pH 7.4, and then blocked for 2 days in modified PB containing 3% donkey serum. After that, retinas were incubated with primary antibody for 5 days and secondary antibody for 2 days at 4°C.

To label retinorecipient regions, 2 μl of cholera toxin B conjugated to Alexa Fluor 488 (CTb-488: 1% in saline; Thermo Fisher Scientific) was injected into each eye. After 24 h, the animal was perfused transcardially with 4% paraformaldehyde, and the brain was removed for post-fixed for 3–5 d at 4°C. After washing with phosphate buffered saline, the brains were sectioned at 80 μm in a cryostat. Immunohistochemistry proceeded as for the retina, except that incubation times with primary and secondary antibody were reduced to 2 and 1 day respectively.

The primary antibodies used were as follows: rabbit anti-RFP (1:1000, Rockland 600-401-379), chicken anti-RFP Biotin conjugated (1:200, Rockland 600-906-379), guinea pig anti-RBPMS (1:500, PhosphoSolutions 1832-RBPMS), mouse anti-AP2 (3 μg/ml, DSHB 3B5), goat anti-Osteopontin (1:500, R&D 441-OP-050), rabbit anti-CART (1:1000, Phoenix pharmaceuticals H-003-62), goat anti-FOXP2 (1:500, Abcam ab1307), rabbit anti-melanopsin (1:1000, Advanced Targeting Systems AB N38), goat anti-choline acetyltransferase (1:500, Millipore AB144P), rabbit anti-GABA (1:2000, Sigma-Aldrich A2052), goat anti- GLYT1 (1:25, Santa Cruz Biotechnology sc-16703), rabbit anti-VIP (1:1000, ImmunoStar 20077), rat anti-SST (1:10, Millipore MAB354). Secondary antibodies were conjugated to Alexa Fluor 488 (Invitrogen), Cy3 (Jackson ImmunoResearch), or Cy5 (Jackson ImmunoResearch). All secondary antibodies were used at a dilution of 1:200.

### Imaging and Morphological Analysis

Images were captured with a Zeiss LSM-510 Meta confocal microscope and processed with LSM Image software, Image J and Photoshop. Flat-mount images of retinas were acquired with either a Plan-Neofluar 25x/0.8 Imm Corr objective or a Plan-Apochromat, 63×/1.4 oil objective. Z-stack images of tdTomato-labeled cells and ChAT antibody-labeled ChAT bands were obtained with a Plan-Apochromat, 63×/1.4 oil objective at 0.25 μm intervals. Brain sections were imaged with a Plan-Neofluar 10x/0.3 air objective, with enlarged views imaged with a Plan-Neofluar 25x/0.8 Imm Corr objective.

To measure the size of the dendritic field, a convex polygon was drawn connecting the outermost tips of the dendrites, and the area within this contour was measured. The diameter of the dendritic field was calculated from the measured area by assuming the dendritic field is circular. Soma diameter was calculated in the same way. To measure the level of stratification of ACs and RGCs, a rectangle representing the region of interest (∼200 μm × 70 μm) was placed near the center of a dendritic field. The image data within the ROI at each level of the *z*-stack was projected onto the *x*-axis. The stratification levels were determined from the upper and lower boundaries of tdTomato-labeled arbors relative to the choline acetyltransferase (ChAT)-positive bands (60 and 27% of the IPL) in XZ plane. For wide-field ACs, the same procedure was repeated on the middle and distal parts of the dendritic field or “axon-like” arbors to verify the consistency of stratification in different parts of the cell. Data are presented as mean ± SD.

## Results

### General Screening Strategy

While Cre drivers have been screened for their expression patterns in the retina (Ivanova et al., 2010; Lu et al., 2013; Zhu et al., 2014; Martersteck et al., 2017), no Flp drivers have been screened. We recently acquired seven Flp drivers: *Pvalb-FlpE*, *Slc32a1-FlpO*, *Sst-FlpO*, *Vip-FlpO*, *Nkx2-FlpO*, *Dlx5/6-FlpE* and *Rorb-FlpO*. Our goals in this study were three-fold: first, to characterize the amacrine and RGC expression patterns in these Flp drivers; second, to validate intersectional strategies in the retina; and third, to establish Cre/Flp combination lines that achieve increased specificity for subsequent anatomical and functional studies.

Our screening proceeded in three steps. The first step was to obtain the expression patterns of FLP in the retina, achieved by crossing Flp drivers with a Cre/Flp double-dependent *Ai65* reporter and a ubiquitous Cre driver (*CMV-Cre*). In the second step, FLP expression in RGCs or ACs was separated based on the differential presence/absence of characteristic vesicular neurotransmitter transporters. Specifically, we took advantage of the fact that glutamate transporter 2 (VGLUT2) is expressed in RGCs but not in ACs (Johnson et al., 2003; Land et al., 2004), whereas the vesicular inhibitory amino acid transporter (VIAAT) is expressed in ACs but not RGCs. Thereby, we isolated RGCs and ACs by crossing the Flp drivers with a *Vglut2-Cre* and *Slc32a1-Cre* drivers respectively. Finally, in the third step, single cell morphologies were analyzed in the RGC group and the AC group, and the cell types were assigned based on published work. If the labeling was too dense to resolve single cell structure, we further crossed Flp drivers with ubiquitous inducible Cre drivers such as *UBC-CreERT2* and *CAGGCre-ER*, and achieved sparse labeling by adjusting the dose of tamoxifen.

### General Expression Patterns of Flp in the Retina

**FIGURE 2:**
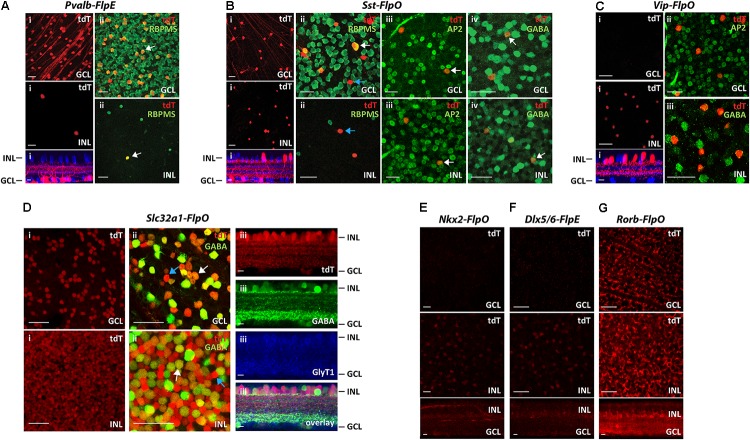
Distribution of FLP-expressing cells in 7 Flp drivers. Each Flp driver was crossed with *CMV-Cre* and *Ai65* mice. **(A)** *Pvalb-FlpE* driver. **(i)** FLPe expressing cells labeled with tdTomato (tdT, red) were observed in the GCL (top) with only a few cells in the INL (middle). Bottom: side view with ChAT (blue). **(ii)** Staining for the RGC marker RBPMS (green) confirmed that all of the tdTomato-labeled cells (tdT, red) in the GCL and the INL were RGCs. White arrows point to example cells that express RBPMS. **(B)** *Sst-FlpO* driver. **(i)** tdTomato-labeled cells were distributed in both the GCL (top) and the INL (middle). Bottom: side view with ChAT (blue).**(ii)** RBPMS staining (green). SST^+^ RGCs were found in the GCL, but not in the INL. White arrow indicates a RBPMS^+^ cell (RGC), blue arrow indicates a RBPMS^-^ cell (presumably an amacrine cell). **(iii)** An amacrine cell marker, AP-2 (green) overlaps with SST^+^ amacrine cells in both the GCL and the INL. White arrows indicate example cells that express AP2. **(iv)** GABA staining (green). White arrows indicate example cells expressing GABA. SST^+^ amacrine cells in both GCL and INL were GABAergic. **(C)** *Vip-FlpO* driver. **(i)** A majority of the targeted cells (red) in the *Vip-FlpO* driver were located in the INL (middle). Bottom: side view with ChAT (blue). These presumptive GABAergic amacrine cells were positive for both AP2 **(ii)** and GABA staining **(iii)**. **(D)** *Slc32a1-FlpO* driver. **(i)** Labeling density was high in both the GCL and INL. **(ii)** Double labeling for tdTomato (red) and GABA (green). In the GCL, ∼80% of cells were GABAergic, whereas in the INL, ∼50% of tdT cells were GABAergic. Examples of GABAergic and non-GABAergic cells (presumably glycinergic amacrine cells) are indicated by white and blue arrows, respectively.**(iii)** Vertical sections showed that tdTomato-labeled cells were either GABAergic (green, GABA staining) or glycinergic (blue, GlyT1 staining). **(E,F)** *Nkx2-FlpO driver***(E)** and *Dlx5/6-FlpE* driver **(F)** showed very little labeling in both the GCL and the INL. **(G)** *Rorb-FlpO* driver showed broad labeling in the GCL and INL, with the strongest labeling in Müller cells. Scale bar, 40 μm for flat-mount view, 10 μm for side view.

The results from seven Flp drivers are summarized in **Figure 2**. Among the seven Flp drivers, the *Pvalb-FlpE* drove FLPe expression mainly in the GCL, with only a few cells in the INL (**Figure 2Ai**). These FLPe-expressing cells were exclusively RGCs, as confirmed by RBPMS immunoreactivity, a marker for RGCs (Rodriguez et al., 2014) (**Figure 2Aii**). The *Sst-FlpO* driver targeted both RGCs and ACs (**Figure 2Bi**). In the GCL, there were both RGCs, confirmed with the RBPMS antibody (**Figure 2Bii**, Top) and GABAergic ACs confirmed with AP2 (Activating protein 2) and GABA antibodies (**Figures 2Biii,iv**, top). AP2 is a family of transcription factors that have been shown to play essential roles in development (Hilger-Eversheim et al., 2000; Eckert et al., 2005). In both mammalian and avian retinas, AP2 is exclusively expressed in postmitotic ACs, but not in other cell types (Bisgrove and Godbout, 1999; Bassett et al., 2007). SST^+^ cells in the INL belonged to GABAergic ACs based on AP2 and GABA staining (**Figures 2Biii,iv**, bottom). Since, both RGCs and ACs were targeted in the *Sst-FlpO* driver, it a good preparation in which to test for the feasibility of RGCs/ACs segregation using the *Vglut2-Cre* or the *Slc32a1-Cre* in intersection (described later). The *Vip-FlpO* driver exclusively targeted ACs (**Figure 2C**). Consistent with the expression pattern of a *Vip-Cre* line (Zhu et al., 2014), the majority of the targeted cells in the *Vip-FlpO* driver were located in the INL (**Figure 2Ci**, bottom). These cells were positive for both AP2 and GABA labeling (**Figures 2Cii,iii**), indicating that they were all GABAergic ACs. The *Slc32a1-FlpO* drove FLPo expression in almost all ACs, hence labeling density was high and widespread (**Figure 2Di**). Slc32a^+^ cells included both the GABAergic and non-GABAergic cells (**Figure 2Dii**). The non-GABAergic cells were glycinergic ACs positively stained with a GLYT1 antibody (**Figure 2Diii**). *Nkx2-FlpO* and the *Dlx5/6-FlpE* had very low expression in retinal cells (**Figures 2E,F**). Finally, *Rorb-FlpO* consistently targeted many types of bipolar cells, ACs, and RGCs, but expression levels were strongest in Müller cells (**Figure 2G**). Based on these results, the *Nkx2-FlpO*, *Dlx5/6-FlpE* and *Rorb-FlpO* drivers were excluded from further analysis.

### Genetic Dissection of Individual Cell Types in Flp Drivers

To provide a benchmark for judging the efficacy of the intersectional approach, we next characterized the cell types labeled in the parent Flp driver lines (**Table 2**) with a focus on *Pvalb-FlpE*, *Sst-FlpO*, and *Vip-FlpO*.

**Table 2:** Summary of AC and RGC types in Flp drivers.

Flp driver	Amacrine cell types	RGC types
*Pvalb-FlpE*	No	on-alpha RGC, off-alpha RGC, ooDSGC, on-DSGC, F-mini-off RGC, F-mini-on RGC, sbcRGC, CRH-3, G1, G4
*Sst-FlpO*	SST-AC1, SST-AC2, SAC	on-alpha RGC, off-alpha RGC, ooDSGC, F-mini-on RGC, F-midi-off RGC, W3, G2, G4, G14, G30
*Vip-FlpO*	VIP-1 AC, VIP-2 AC, VIP-3 AC	No
*Slc32a1-FlpO*	More than 95% of ACs	Very few
*Rorb-FlpO*	Many types	Many types
*Nkx2-FlpO*	Low expression	Low expression
*Dlx5/6-FlpE*	Low expression	Low expression

#### The *Pvalb-FlpE* Driver Targets at Least 10 Types of RGCs

**FIGURE 3:**
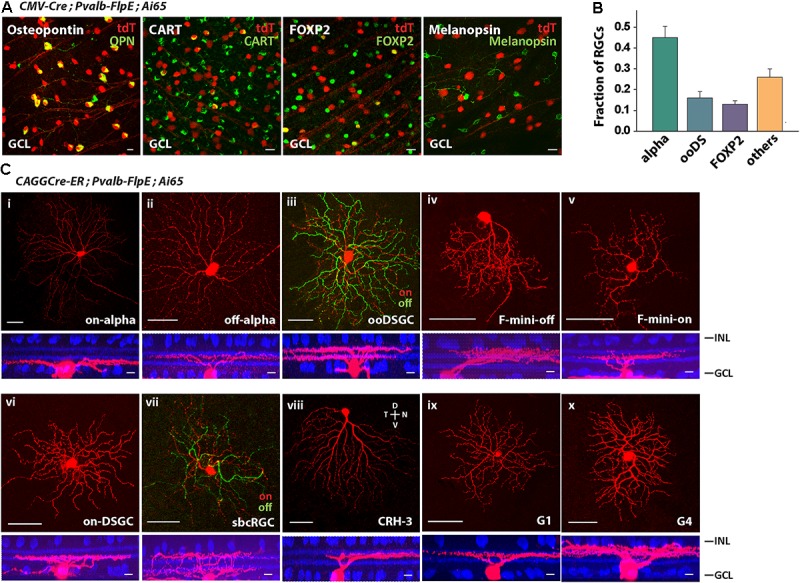
The *Pvalb-FlpE* driver targeted multiple RGC types. **(A)** The *Pvalb-FlpE* driver was crossed with *CMV-Cre* and *Ai65* mice. Immunostainings were performed by using antibody markers for 4 different RGC types: Osteopontin for alpha RGCs, CART for ooDSGCs, FOXP2 for F-RGCs, and melanopsin for ipRGCs. Scale bar, 20 μm. **(B)** Proportions of RGC types targeted in the *Pvalb-FlpE* driver. *n* = 6 retinas from 6 animals (6 litters). **(C)** RGC types extracted from *CAGGCre-ER;Pvalb-FlpE;Ai65* retinas without tamoxifen administration. Flat-mount view (top) and side view (bottom) with ChAT (blue) labeling. Scale bar: 50 μm for the flat-mount view, 10 μm for the side view.

The most efficient way to survey cell types is to use type-specific molecular markers. We labeled PVALB^+^ RGCs with molecular markers for the following RGCs (Rousso et al., 2016): Osteopontin for alpha RGCs, CART for ooDSGCs (on-off DSGCs), FOXP2 for F-RGCs, and melanopsin for ipRGCs (**Figure 3A**). Forty-five percent of PVALB^+^ RGCs were alpha RGCs, 16% were ooDSGCs, 13% were F-RGCs, and none were melanopsin positive ipRGCs (**Figure 3B**). About 26% of PVALB^+^ RGCs were not labeled by any of the four antibodies. In order to define the subtypes of the unlabeled cells, we applied established cell morphological criteria. We crossed the Pvalb-FlpE driver with a CAGGCre-ER driver and Ai65 reporter line. The CAGGCre-ER driver has a small amount of “leaky” Cre expression in the absence of tamoxifen, which allowed us to visualize individual cells for single-cell reconstruction. In addition to alpha RGCs, ooDSGCs, F-RGCs (**Figures 3Ci–v**), the analysis of single cell morphologies revealed that Pvalb-FlpE targeted at least 5 more RGC types that resemble on-DSGCs, sbcRGCs, CRH-3 RGCs (Zhu et al., 2014), G1 RGCs and G4 RGCs (Baden et al., 2016). Examples of single cell morphologies representing each RGC type are shown in **Figures 3Cvi–x**. It has been reported that the Pvalb-Cre driver shows differential labeling across the retina (Baden et al., 2016). In this study, the cells were analyzed across the entire retina instead of any preferred locations.

#### The Sst-FlpO Driver Targets at Least 10 Types of RGCs and 3 Types of ACs

**FIGURE 4:**
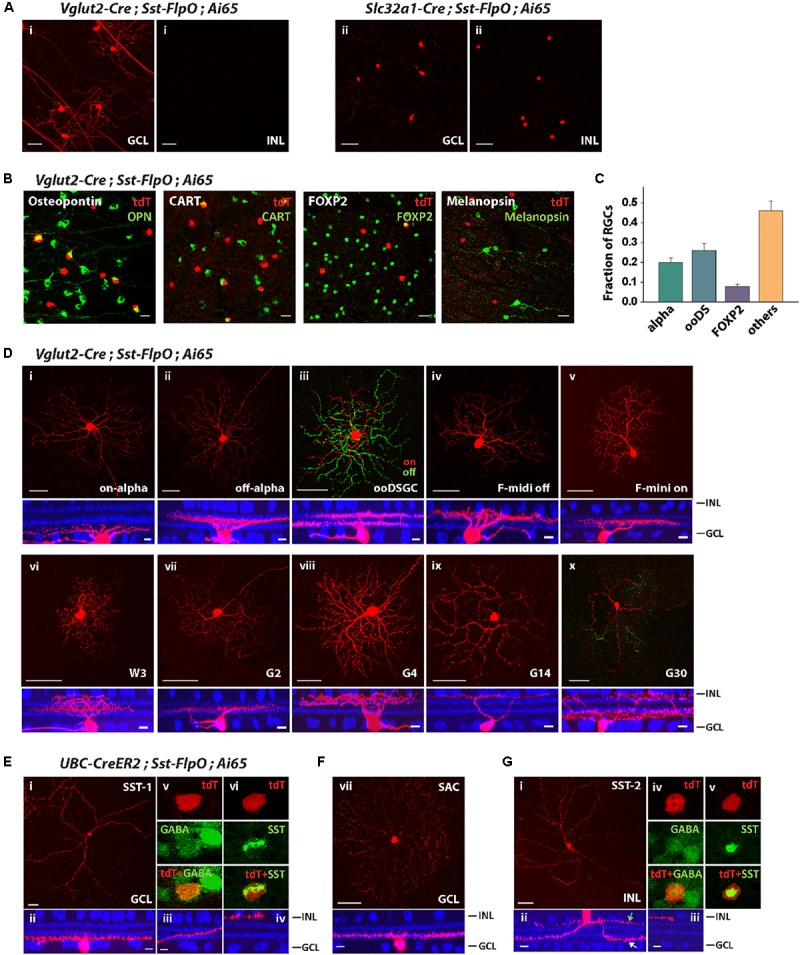
The *Sst-FlpO* driver targeted both RGCs and ACs. **(A)** **(i)** To identify targeted RGCs, the *Sst-FlpO* driver was crossed with *Vglut2-Cre* and *Ai65* reporter mice. **(ii)** To identify targeted amacrine cells, the *Sst-FlpO* driver was crossed with *Slc32a1-Cre* and *Ai65* mice. Scale bar, 50μm. **(B)** Staining for RGC markers (green). RGC constituents were probed with antibodies against Osteopontin, CART, FOXP2 and melanopsin in the *Vglut2-Cre*;*Sst-FlpO;Ai65* retinas. Scale bar, 20 μm. **(C)** Proportions of RGC types targeted in the *Sst-FlpO* driver. *n* = 7 retinas from 7 animals (6 litters). **(D)** Individual RGC types extracted from the *Vglut2-Cre*;*Sst-FlpO;Ai65* retinas. Flat-mount views (top) and side views (bottom) with ChAT (blue). Scale bar: 50 μm for the flat-mount view, 10 μm for the side view. **(E–G)** Amacrine cell types extracted from the *UBC-CreER2;Sst-FlpO;Ai65* retinas, without tamoxifen administration. **(E)** Representative images of an SST-1 AC. **(i)** Flat-mount view of the soma and surrounding processes. **(ii)** Side view of the soma and surrounding “dendrite-like” and “axon-like” processes. **(iii)** Axon-like processes traveled across the IPL and ended at the INL border **(iv)**. An SST-1 AC was co-labeled with antibodies against GABA **(v)** and SST **(vi)**. **(F)** A starburst amacrine cell (SAC) in the GCL. Flat-mount view (top) and side view (bottom) with ChAT (blue). **(G)** SST-2 AC. Flat-mount view of the soma and surrounding processes. **(ii)** Side view of the soma and surrounding dendrite-like (white arrow) and axon-like processes (green arrow). **(iii)** Axon-like process end at the INL border. An SST-2 AC was co-labeled with antibodies against GABA **(iv)** and SST **(v)**. Scale bar for **(E–G)**: 50 μm for the flat-mount view, 10 μm for the side view.

Since the Sst-FlpO driver expressed transgenes in both RGCs and ACs (**Figure 2B**), we tested whether we could separate RGCs from ACs by crossing Sst-FlpO mice with Vglut2-Cre and Slc32a-Cre mice, respectively. As predicted, all the AC labeling was removed and only RGCs remained labeled in the Vglut2-Cre/Sst-FlpO cross (**Figure 4Ai**). The labeled RGCs were spaced far apart, enabling us to perform single-cell reconstruction. In the Slc32a-Cre/Sst-FlpO cross, only ACs remained labeled, including cells in both INL and GCL (**Figure 4Aii**). AC labeling was dense and required further segregation.

To characterize the distribution of RGC types in the *Vglut2-Cre*/*Sst-FlpO* retina, we first labeled SST^+^ RGCs with antibodies against Osteopontin, CART, FOXP2 and melanopsin (**Figure 4B**). Unlike in the *Pvalb-FlpE* driver, alpha RGCs only accounted for 20% of SST^+^ RGCs, ooDSGCs accounted for 26%, and F-RGCs accounted for 8% of SST^+^ RGCs (**Figure 4C**). No SST^+^ RGCs was labeled by the antibody to melanopsin. Forty-six percent of SST^+^ RGCs were unidentified by the antibodies. Single-cell reconstruction revealed that, in addition to alpha RGCs, ooDSGCs, and F-RGCs (**Figures 4Di–v**), the *Sst-FlpO* driver targeted at least 5 more RGC types. Their morphologies strongly resembled those of W3, G2, G4, G14, and G30 (Baden et al., 2016) (**Figures 4Dvi–x**). Although alpha RGCs were the dominant RGC in the *Pvalb-FlpE* driver, RGCs in the *Sst-FlpO* driver were more evenly distributed among at least 10 RGC types.

Since AC labeling was still quite dense in the *Slc32a-Cre*/*Sst-FlpO* line, we identified ACs in the *Sst-FlpO* driver by crossing it with an inducible Cre driver, *UBC-CreERT2*, which facilitated sparse labeling. We ignored labeled RCGs in the *UBC-CreERT2*/*Sst-FlpO* retina and instead identified three types of ACs, two in the GCL and one in the INL. The first AC type in the GCL was similar to the SST-1 AC identified in the *Sst-Cre* driver (Zhu et al., 2014). It had a soma ∼13 μm (12.8 ± 1.3 μm) in diameter and gave rise to thick “dendrite-like” processes and a long, fine axon-like process (**Figure 4Ei**). The dendrite-like arbors ramified at the GCL border (**Figure 4Eii**), while the axon-like arbor started off near the GCL border and then traversed the width of the IPL and ended in a ramification at the INL border (**Figures 4Eii–iv**). SST-1 ACs were GABAergic (**Figure 4Ev**) and labeled with an antibody to SST (**Figure 4Evi**). The second AC type in the GCL was a displaced starburst AC (**Figure 4F**), which was also observed in the *Sst-Cre* driver (Zhu et al., 2014). The AC type in the INL (named SST-2 AC) had a soma around 11 μm (10.9 ± 1.2 μm) in diameter, and gave rise to 2–3 dendrite-like processes (**Figure 4Gi**) that branched and ramified at the GCL border (**Figure 4Gii**), and 1-2 axon-like processes that remained at the INL border (**Figure 4Giii**). SST-2 ACs were GABAergic (**Figure 4Giv**) and positive for SST antibody labeling (**Figure 4Gv**).

#### The *Vip-FlpO* Driver Targets 3 Types of ACs

**FIGURE 5:**
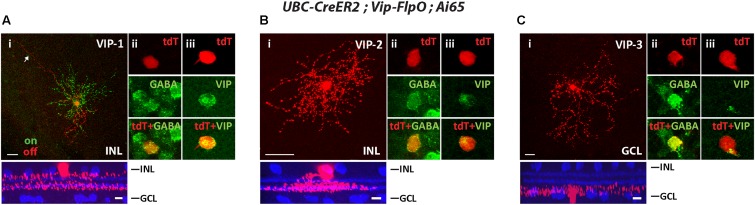
The *Vip-FlpO* driver targeted 3 types of ACs. The *Vip-FlpO* driver was crossed with *UBC-CreER2* and *Ai65*, 20 μg tamoxifen was administered to achieve sparse labeling of ACs. Representative images for VIP-1 AC **(A)**, VIP-2 AC **(B)**, and VIP-3 AC **(C)** are shown in flat-mount view (top) and side view (bottom) with ChAT (blue) in **(i)**. Arrow in **(Ai)** shows the “tail” of the VIP-1 AC. Sections were co-labeled with antibodies against GABA (green) **(ii)** and VIP (green) **(iii)**. Scale bar: 25 μm for flat-mount view, 10 μm for side view.

To segregate cell types in the Vip-FlpO driver, we crossed it with the UBS-CreERT2 driver and achieved sparse labeling with a low dose of tamoxifen. The Vip-FlpO driver targeted FLPo expression in three AC types, consistent with the Cre expression pattern described in the Vip-Cre driver (Akrouh and Kerschensteiner, 2015; Park et al., 2015; Perez de Sevilla Muller et al., 2017). The first type was a wide-field bistratified AC previously named VIP-1 (Zhu et al., 2014; Akrouh and Kerschensteiner, 2015). VIP-1 ACs have somata (11.2 ± 1.1 μm in diameter) located in the INL with dendrites that ramified in two layers (**Figure 5Ai**): the ON dendrites ramified between the ON ChAT band and the GCL (75.8% ± 3.5% of the IPL) and the OFF dendrites ramified between the OFF ChAT band and the INL (12.9% ± 1.6% of the IPL). Many of the cells had a long process or “tails” that extended at least 200 μm from the soma (**Figure 5Ai**). The ON and OFF dendritic fields (excluding the “tails”) were 201 ± 27 μm and 178 ± 36 μm in diameter, respectively. The second type was a monostratified AC named VIP-2 in previous studies (Akrouh and Kerschensteiner, 2015; Perez de Sevilla Muller et al., 2017). The cell soma was located in the INL and its dendrites ramified in a thick band between 41.8 ± 3.5% and 65.8% ± 0.2% in the IPL covering the ON ChAT band (**Figure 5Bi**). The dendritic field of the VIP-2 AC was 100 ± 21 μm in diameter. Unlike VIP-1 and VIP-2 ACs, the third AC type (named VIP-3 (Akrouh and Kerschensteiner, 2015; Perez de Sevilla Muller et al., 2017)) was a displaced AC with its soma located in the GCL. Its dendrites ramified near the GCL (**Figure 5Ci**). The dendritic field of the VIP-3 AC was 221 ± 43 μm in diameter. All three types of VIP ACs were GABAergic (**Figures 5Aii,Bii,Cii**) and labeled with an antibody to VIP (**Figures 5Aiii,Biii,Ciii**). **Table 2** summaries the AC and RGC types in Flp drivers.

### Further Intersectional Strategies to Confine Retinal Cell Subpopulations

**FIGURE 6:**
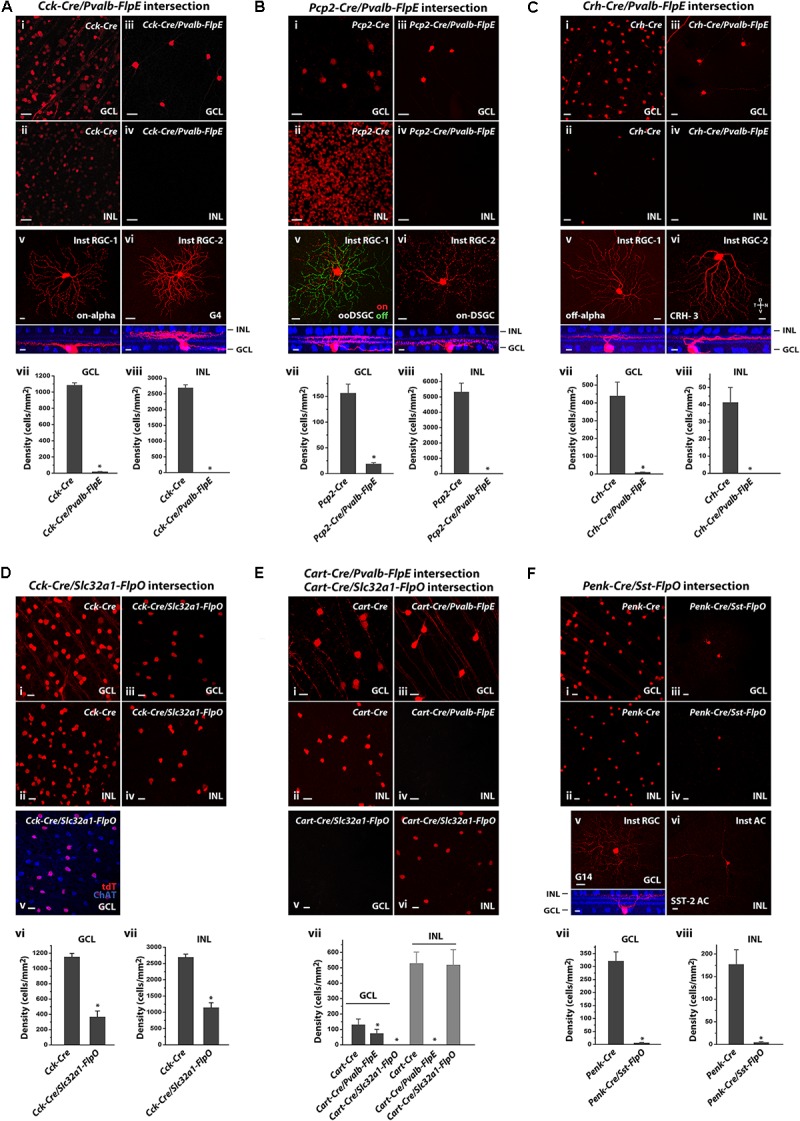
Intersectional strategies to restrict labeling of retinal cell subpopulations. **(A)** *Cck-Cre* driver alone and in intersection with the *Pvalb-FlpE* driver. **(i,ii)** Distribution of tdTomato-labeled cells in the GCL **(i)** and the INL **(ii)** in the *Cck-Cre* driver crossed with the *Ai9* reporter line. **(iii,iv)** Distribution of tdTomato-labeled cells from the *Cck-Cre;Pvalb-FlpE;Ai65* intersection. **(v,vi)** Examples of individual cell types within the *Cck/Pvalb* intersection. Flat-mount view (top) and side view (bottom) with ChAT (blue). **(vii,viii)** Cell labeling density in the *Cck-Cre* and the *Cck-Cre/Pvalb-FlpE* intersection for the GCL **(vii)** or the INL **(viii)**. *n* = 6 retinas from 6 animals (4 litters) for the *Cck-Cre;Ai9. n* = 6 retinas from 6 animals (5 litters) for the *Cck-Cre;Pvalb-FlpE;Ai65.* ^∗^*p* < 0.05, two-tailed *t*-test. **(B)** *Pcp2-Cre* driver alone and in intersection with the *Pvalb-FlpE* driver. **(i,ii)** labeling of *Pcp2-Cre* mice by crossing with the *Ai9* reporter. **(iii,iv)** Intersection of *Pcp2-Cre* and *Pvalb-FlpE* in the *Ai65* reporter. **(v,vi)** Flat mount views (top) and side views (bottom) of the cell types within the *Pcp2/Pvalb* intersection. **(vii,viii)** Cell labeling density in the *Pcp2-Cre* and the *Pcp2-Cre/Pvalb-FlpE* intersection for the GCL **(vii)** or the INL **(viii)**. *n* = 6 retinas from 6 animals (4 litters) for the *Pcp2-Cre;Ai9. n* = 6 retinas from 6 animals (6 litters) for the *Pcp2-Cre;Pvalb-FlpE;Ai65*. ^∗^*p* < 0.05, two-tailed *t*-test. **(C)** *Crh-Cre* driver alone and in intersection with the *Pvalb-FlpE* driver. **(i,ii)** *Crh-Cre* driver expression as reported by *Ai9* mice. **(iii,iv)** Intersection of *Crh-Cre* and *Pvalb-FlpE* in the *Ai65* reporter. **(v,vi)** Example images of flat mount views (top) and side views (bottom) of the cells within the *Crh/Pvalb* intersection. **(vii,viii)** Comparing cell labeling density in the *Crh-Cre* and the *Crh-Cre/Pvalb-FlpE* intersection for the GCL **(vii)** or the INL **(viii)**. *n* = 7 retinas from 7 animals (5 litters) for the *Crh-Cre;Ai9. n* = 7 retinas from 7 animals (5 litters) for the *Crh-Cre;Pvalb-FlpE;Ai65*. ^∗^*p* < 0.05, two-tailed *t*-test. **(D)** *Cck-Cre* driver alone and *Cck-Cre/Slc32a1-FlpO* intersection. **(i,ii)** *Cck-Cre* driver crossed with the *Ai9* reporter. **(iii,iv)** Intersection of *Cck-Cre* and *Slc32a1-FlpO* reported in *Ai65* mice. **(v)** *Cck-Cre/Slc32a1-FlpO* intersection (red) in the GCL counter-stained with an antibody against ChAT (blue). **(vi,vii)** Cell labeling density in the *Cck-Cre* and the *Cck-Cre/Slc32a1-FlpO* intersection for the GCL **(vi)** or the INL **(vii)**. *n* = 6 retinas from 6 animals (5 litters) for the *Cck-Cre;Ai9. n* = 6 retinas from 6 animals (5 litters) for the *Cck-Cre;Slc32a1-FlpO;Ai65*. ^∗^*p* < 0.05, two-tailed *t*-test. **(E)** *Cart-Cre* driver alone and in intersection with *Pvalb-FlpE* or *Slc32a1-FlpO*. **(i,ii)** *Cart-Cre* driver with *Ai9* reporter. **(iii,iv)** *Cart-Cre/Pvalb-FlpE* intersection removed labeling from amacrine cells. **(v,vi)** *Cart-Cre/Slc32a1-FlpO* intersection removed labeling from RGCs. **(vii)** Cell labeling density in *Cart-Cre* alone, *Cart-Cre/Pvalb-FlpE* intersection, and *Cart-Cre/Slc32a1-FlpO* intersection for the GCL (dark gray) or the INL (gray). *n* = 8 retinas from 8 animals (5 litters) for the *Cart-Cre;Ai9. n* = 8 retinas from 8 animals (6 litters) for the *Cart-Cre;Pvalb-FlpE;Ai65. n* = 8 retinas from 8 animals (6 litters) for the *Cart-Cre;Slc32a1-FlpO;Ai65*. ^∗^*p* < 0.05, two-tailed *t*-test. **(F)** *Penk-Cre* driver alone and *Penk-Cre* /*Sst-FlpO* intersection. **(i,ii)** *Penk-Cre* driver with *Ai9* reporter. **(iii,iv)** Intersection of *Penk-Cre* and *Sst-FlpO* in the *Ai65* reporter. **(v,vi)** Example images of individual cell types within the *Penk/Sst* intersection. Flat-mount view (top) and side view (bottom) with ChAT (blue). **(vii,viii)** Cell labeling density in the *Penk-Cre* and the *Penk/Sst* intersection for the GCL **(vii)** or the INL **(viii)**. *n* = 7 retinas from 7 animals (5 litters) for the *Penk-Cre;Ai9. n* = 7 retinas from 7 animals (5 litters) for the *Penk-Cre;Sst-FlpO;Ai65.*^∗^*p* < 0.05, two-tailed *t*-test. Scale bar, 20 μm for flat-mount view, 10 μm for side view.

To validate the feasibility of using Cre/Flp intersections to limit expression to a small subset or even one neuronal type in the retina, we selectively crossed pairs of Cre and Flp drivers showing overlap in expression patterns of RGCs or ACs (**Figure 6** and **Table 3**).

**Table 3:** Intersectional strategies to restrict retinal cell subpopulations.

Flp driver	Bipolar cells	Amacrine cells	RGCs
*Cck-Cre*	Yes	Yes	ooDSGCs, sbcRGCs, on-alpha RGCs, etc.
*Cck-Cre/ Pvalb-FlpE*	No	No	on-alpha RGCs (80%), G4 (20%)
*Pcp2-Cre*	Yes	No	m-BGCs , s-BGCs, b-BGCs, on-DSGCs and ooDSGCs
*Pcp2-Cre/Pvalb-FlpE*	No	No	ooDSGCs (2/3), on-DSGCs (1/3)
*Crh-Cre*	No	CRH-1 AC, CRH-2 AC, CRH-3 AC	off-alpha RGCs, JAM-B RGCS, CRH-3 RGCs
*Crh-Cre/ Pvalb-FlpE*	No	No	off-alpha RGCs (80%), CRH-3 RGCs (20%)
*Cck-Cre*	Yes	Yes	ooDSGCs, sbcRGCs, on-alpha RGCs, etc.
*Cck- Cre/Slc32a-FlpO*	No	Yes	No
*Cart-Cre*	No	Yes	ooDSGCs
*Cart-Cre/Pvalb-FlpE*	No	No	ooDSGCs
*Cart-Cre/Slc32a1-FlpO*	No	Yes	No
*Penk-Cre*	No	Multiple types	Multiple types
*Penk-Cre/Sst-FlpO*	No	SST-2 AC	G14

#### *Pvalb-FlpE* Driver for Targeting Specific RGC Types

Since the *Pvalb-FlpE* driver exclusively targeted RGC types, we reasoned that this line could provide a useful filter to remove non-RGC types in the Cre/Flp intersection. This idea was demonstrated in several examples. The first example was a *Cck-Cre*/*Pvalb-FlpE* intersection. In the *Cck-Cre* line, Cre expression was observed in bipolar, amacrine, ganglion, and Müller cells (Zhu et al., 2014). Bipolar cells made up more than half of the labeled cells in the INL, with the rest being ACs and a small number of Müller cells (**Figure 6Aii**). The GCL contained both labeled ACs and RCGs (**Figure 6Ai**) including the following types: starburst ACs, ooDSGCs, sbcRGCs, on-alpha RGCs (Zhu et al., 2014), and other RGC types. Thus, combining the *Cck-Cre* and *Pvalb-FlpE* drivers should allow us to isolate the CCK/PVALB-double positive RGCs. Indeed, the *Cck-Cre*/*Pvalb-FlpE* combination effectively removed all the labeling from the INL (**Figures 6Aiv,viii**), as well as 98% of the labeling in the GCL (**Figures 6Aiii,vii**). Only two types of RGCs remained labeled: one type (80% of the sample) resembled on-alpha RGCs (**Figure 6Av**) and the other type (20% of the sample) resembled G4 (Baden et al., 2016) (**Figure 6Avi**).

In a second example, we created a *Pcp2-Cre*/*Pvalb-FlpE* intersection. The *Pcp2-Cre* driver predominantly targets bipolar cells (Lu et al., 2013) along with five types of RGCs: m-BGCs, s-BGCs, b-BGCs, on-DSGCs and ooDSGCs (Ivanova et al., 2013). Our analysis of *Pcp2-Cre* expression (**Figures 6Bi,ii**) is in agreement with previous reports. As expected, bipolar cell labeling was absent in the *Pcp2-Cre*/*Pvalb-FlpE* cross (**Figures 6Biv,viii**). Additionally, this combination also removed labeling from three out of five RGC types (m-BGCs, s-BGCs, b-BGCs) leaving only two types labeled: on-DSGCs and ooDSGC (**Figures 6Biii,vii**): two-thirds were on-off DSGCs (**Figure 6Bv**) versus one-third on-DSGCs (**Figure 6Bvi**).

In a third example, we crossed the *Pvalb-FlpE* and *Crh-Cre* drivers to isolate a population of CRH/PVALB-double positive RGCs. As demonstrated in previous studies, the *Crh-Cre* driver targeted at least three types of ACs (Park et al., 2018) and three types of RGCs in the GCL (off-alpha RGCs, JAM-B RGCS and CRH-3 RGCs) (Zhu et al., 2014), as well as a small number of ACs in the INL (**Figures 6Ci,ii**). The *Crh-Cre*/*Pvalb-FlpE* intersection removed labeling from the ACs in the GCL (**Figures 6Ciii,vii**) and the INL (**Figures 6Civ,viii**). Eventually, only two types of RGCs remained: more than 80% of the labeled cells belonged to off-alpha RGCs (CRH-1 RGCs, **Figure 6Cv**) while the rest were CRH-3 RGCs (**Figure 6Cvi**) (Zhu et al., 2014).

#### *Slc32a1-FlpO* Driver for Targeting AC Types

In contrast to the *Pvalb-FlpE* driver, the *Slc32a1-FlpO* driver only targeted ACs thus it could be used to remove labeling from both bipolar cells and RGCs. To test the effectiveness of the *Slc32a1-FlpO* driver in this application, we paired it with the *Cck-Cre* driver which broadly targets bipolar cells, ACs, Müller cells and RGCs (**Figures 6Di,ii**). As predicted, crossing *Slc32a1-FlpO* with *Cck-Cre* effectively removed labeling from bipolar and Müller cells in the INL, and only AC labeling remained (**Figures 6Div,vii**). In the GCL, all the RGC labeling was removed (**Figures 6Diii,vi**) and the remaining labeled ACs were all starburst as confirmed with ChAT antibody staining (**Figure 6Dv**), consistent with previous results (Zhu et al., 2014).

The foregoing experiments demonstrated intersectional applications of two Flp drivers: *Pvalb-FlpE* and *Slc32a1-FlpO* with each crossed with different Cre drivers. Next, we reasoned that, if we applied *Pvalb-FlpE* intersection and *Slc32a1-FlpO* intersection in parallel, we could separately target ACs and RGCs in a same Cre driver for functional study. Here we chose the *Cart-Cre* driver. CART (Cocaine and amphetamine-regulated transcript) expresses in 15% of all RGCs as well as in a small group of ACs in the inner nuclear layer. It has been demonstrated that all CART-expressing RGCs are ooDSGCs (Kay et al., 2011; Park et al., 2014). In the *Cart-Cre* driver, all the Cre-positive cells in the GCL were ooDSGCs, while the cells in the INL belonged to two types of ACs (**Figures 6Ei,ii**).

Crossing the *Cart-Cre* with the *Pvalb-FlpE* eliminated all the AC labeling (**Figures 6Eiv,vii**), leaving only ooDSGCs labeling in the GCL (**Figure 6Eiii**). On the other hand, crossing the *Cart-Cre* with the *Slc32a-FlpO* removed all the labeling from ooDSGCs (**Figures 6Ev,vii**), leaving only AC labeling in the INL (**Figure 6Evi**). This result demonstrates that intersectional strategies with the *Pvalb-FlpE* and *Slc32a1-FlpO* drivers can effectively lead to highly selective cell type-specific targeting in Cre lines driving expression in both ACs and RGCs.

#### *Sst-FlpO* Driver for More Specific Targeting of AC and RGC Types

The *Sst-FlpO* driver targeted only three types of ACs. We reasoned if we could find a suitable Cre driver to pair with the *Sst-FlpO* driver, we could potentially reduce the number of targeted AC types. We selected the *Penk-Cre* driver which targeted Cre expression to multiple types of ACs in the INL (**Figure 6Fii**) and multiple types of ACs and RGCs in the GCL (**Figure 6Fi**). Interestingly, we found that when the *Penk-Cre* driver was crossed with *SST-FlpO* driver, highly specific targeting was achieved as the *Penk-Cre*/*Sst-FlpO* intersection mostly captured one type of ACs in the INL (**Figures 6Fiv,viii**) that appeared to be the SST-2 AC (**Figure 6Fvi**) and one type of RGCs in the GCL (**Figures 6Fiii,vii**) that resembled G14 RGC (**Figure 6Fv**) (Baden et al., 2016), although the labeling was rather sparse. **Table 3** summarizes the AC and RGC types obtained from the intersectional strategies.

### Screening of Flp Drivers for Their Expression in Retino-Recipient Regions

**FIGURE 7:**
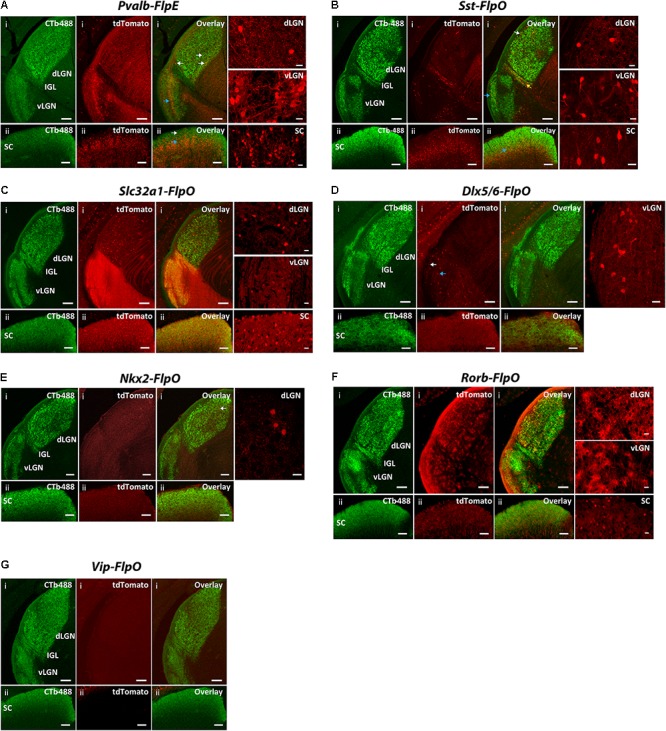
Expression patterns of Flp drivers in the SC and the LGN. FLP-expressing cells were labeled with tdTomato (red) in *CMV-Cre;Flp;Ai65* mice. Retino-recipient zones were labeled with cholera toxin B conjugated to Alexa Fluor 488 (CTb-488) (green) injected to the eyes. **(A)** *Pvalb-FlpE* driver. In the SC, FLPe strongly expressed in the deep layer (**ii**, blue arrow) but sparsely expressed in the superficial layer (**ii**, white arrow). The middle layer of vLGN also strongly expressed FLPe (**i**, blue arrow). The dLGN showed very sparse labeling (**i**, white arrows). Intense tdTomato background comes from axons of FLPe^+^ RGCs. **(B)** *Sst-FlpO* driver. Strong FLPo expression was found in the deep layer of the SC (**ii**, blue arrow). Only sparse labeling was found in the vLGN and dLGN, while most of the labeled cells were in the superficial layer (**i**, white arrow for dLGN, blue arrow for vLGN). There was also moderate labeling in IGL (yellow arrow). **(C)** *Slc32a1-FlpO* targeted FLPo expression broadly to all the layers in the LGN and SC. **(D)** *Dlx5/6-FlpO* driver. FLPo expression was sparse and restricted primarily to the superficial (**i**, white arrow) and middle layer of the vLGN (**i**, blue arrow). No FLPo labeling was found in SC and dLGN. **(E)** *Nkx2-FlpO* driver. In the dLGN, sparely labeled cells were found in the core region (**i**, arrow). There was no labeling in other areas. **(F)** *Rorb-FlpO* driver. FLPo expression was observed in neurons in all layers in the SC **(ii)**, as well as glial cells in the entire LGN including both the dLGN and the vLGN **(i)**. **(G)** *Vip-FlpO* driver did not express in either the LGN or the SC. For each driver, examples of enlarged labeling area are shown on the right. Scale bar: 100 μm for the LGN, 50 μm for the SC, 20 μm for the enlarged regions.

Finally, we examined FLP expression in the seven drivers in two major retino-recipient regions in the brain: the lateral geniculate nucleus (LGN) and the superior colliculus (SC) (**Figure 7** and **Table 4**). FLP-expressing cells were labeled with tdTomato in *CMV-Cre*/*Flp*/*Ai65* mice. To mark retino-recipient zones, we performed intraocular injection of cholera toxin B conjugated to Alexa Fluor 488 (CTb-488), which fills RGC terminals via anterograde transport.

**Table 4:** FLP expression in the dLGN and the SC in Flp drivers.

Flp driver	dLGN	SC
*Pvalb-FlpE*	shell (+), core (+)	Deep layer (*++)*, superficial layer (+)
*Sst-FlpO*	shell (+)	Deep layer (++)
*Slc32a1-FlpO*	shell (++), core (++)	Deep layer (++), superficial layer (++)
*Rorb-FlpO*	Glial cells	Deep layer (++), superficial layer (++)
*Nkx2-FlpO*	Core (+)	None
*Dlx5/6-FlpE*	None	None
*Vip-FlpO*	None	None

*++, Strong expression; +, sparse expression*.

In the *Pvalb-FlpE* driver, we observed prominent FLP expression in the deep layer of the SC but sparse expression in the superficial layer of the SC (**Figure 7Aii**). The middle layer of the vLGN also showed strong labeling (**Figure 7Ai**). However, only very sparse labeling was detected in both the shell and the core of the dLGN (**Figure 7Ai**). Note that *Pvalb-FlpE* targeted many RGCs whose axonal processes contributed to the strong tdTomato background in the image shown. In the *Sst-FlpO* driver, strong FLPo expression was found in the deep layer of the SC (**Figure 7Bii**). Sparse labeling was found in the vLGN and dLGN, with most of the labeled cells located in the superficial layer (**Figure 7Bi**). Interestingly, there was moderate labeling in the IGL in the *Sst-Flp* driver (**Figure 7Bi**). In the *Slc32a1-FlpO* driver, strong FLPo expression was observed in all layers in the dLGN, vLGN, and SC (**Figures 7Ci,ii**). In the *Dlx5/6-FlpE* driver, FLPe expression was restricted to the superficial and middle layers of the vLGN (**Figure 7Di**). No FLPo labeling was found in the SC and the dLGN (**Figures 7Di,ii**). In the *NKx2-FlpO* driver, neurons expressing the tdTomato reporter were only detected in the dLGN where they were sparely distributed within the core region (**Figure 7Ei**), no FLPo expression was detected in the SC (**Figure 7Eii**). In the *Rorb-FlpO* driver, FLPo expression targeted neurons in the SC (**Figure 7Fii**) and glial cells in the entire LGN including both the dLGN and the vLGN (**Figure 7Fi**). In the *Vip-FlpO* driver, no FLPo expression was observed in either the LGN or the SC (**Figure 7G**).

In summary, the SC labeling was observed from four lines: *Pvalb-FlpO* (all layers), *Sst-FlpO* (deep layer), *Slc32a1-FlpO* (all layers) and *Rorb-FlpO* (all layers). Meanwhile, dLGN labeling was observed in four lines: *Pvalb-FlpE* (shell and core), *Sst-FlpO* (shell), *Slc32a1-FlpO* (shell and core) and *NKx2-FlpO* (core). Finally, vLGN labeling was observed in four lines: *Pvalb-FlpE* (middle layer), *Sst-FlpO* (superficial layer) *Slc32a1-FlpO* (all layers) and *Dlx5/6-FlpE* (superficial layer and middle layer).

## Discussion

As in many other brain circuits, there are two major challenges for dissecting retinal circuits: (Demb and Singer, 2015) to increase the specificity of cell-type targeting and (Masland, 2012) to discover new cell types involved in the circuits. Here we used an intersectional strategy, specifically the combination of Cre/LoxP and Flp/FRT systems, to increase the specificity for targeting amacrine and ganglion cell types in the mouse retina. The Flp-FRT system carries out site-specific recombination similar to the Cre-loxP system, however the efficiency of recombination mediated by the wild type Flp recombinase is lower than that of Cre. Consequently Cre/loxP was initially chosen as the main genetic site-specific recombination system, leading to the development of hundreds of Cre driver lines over the past two decades (Gong et al., 2007). To improve the Flp-FRT system, a thermostable derivative of wild type Flp, FlpE (enhanced Flp), was developed and was further codon-optimized (named FlpO) (Bertolotto et al., 2016; Euler and Baden, 2016). These improvements made the Flp/FRT and Cre/loxP systems equally efficient for mouse genetic manipulations. Consequently, the last several years have seen the creation of increasing numbers of Flp lines, a trend that is expected to continue. Thus, we expect that strategies that we have tested here will have even greater applicability in the future.

Two parallel approaches could be used to facilitate the identification of suitable pairs of Cre and Flp drivers for intersection. First, information from transcriptome studies and protein expression profiling studies can be used to identify gene specific markers for retinal cell types/subtypes. In recent years, RNA sequencing (RNA-Seq)-based transcriptome analysis has begun to transform the study of the gene expression in the retina field (Siegert et al., 2012; Macosko et al., 2015; Shekhar et al., 2016; Rheaume et al., 2018). *In silico* data mining will allow us to select candidate genes whose promoters can be used to construct appropriate Cre lines and Flp lines. However, these mice are likely not available yet. In addition, due to the complex nature of gene expression, it is often unsurprising that transgene expression in a given transgenic mouse line or knock-in mouse line differ from endogenous gene expression. An alternative approach is to screen the available Cre and Flp drivers, and then select the appropriate pair with overlapped cell types. Given the limited information about the genetic profiling of many retinal cells and the limited number of available Flp drivers, we think the most straightforward approach at this stage is to screen the available Flp drivers so as to provide a base for future intersectional crossing with the appropriate Cre lines.

Since an intersectional approach is relatively new in retinal research, it is important to validate the feasibility of this strategy for targeting subpopulations of retinal cell types. Our experiments demonstrated two major advances for targeting retinal cells. First, intersections can be used to eliminate (or select for) whole classes of cells (i.e., ACs or RCGs). For example, the *Pvalb-FlpE* driver exclusively targets RGC types, thus it can be used to remove labeling from non-RGC types in the Cre/Flp intersection. In this study, we crossed *Pvalb-FlpE* lines with *Cck-Cre*, *Pcp2-Cre*, *Crh-Cre* and *Cart-Cre* drivers. In all cases, we found that only RGCs were selectively labeled while non-RGC labeling was removed. Conversely, we selected a *Slc32a1-FlpO* driver to eliminate non-AC labeling as the *Slc32a1-FlpO* driver only targeted ACs. Indeed when the *Slc32a1-FlpO* driver was paired with a *Cck-Cre* driver or a *Cart-Cre* driver, all non-AC labeling was effectively removed. Thus we have demonstrated that an intersectional strategy works to eliminate labeling from whole classes of cells. It is important to note that while *Pvalb-FlpE* only targets certain RCG types, it does not cover all of the RGCs. Therefore crossing Cre drivers with *Pvalb-FlpE* will filter out not only the non-RGC cells but also many RGCs not covered by *Pvalb-FlpE*. The *Slc32a1-FlpO* driver, on the other hand, targets more than 95% of ACs. Thus, a vast majority of the ACs targeted in Cre drivers will remain when crossed with the *Slc32a1-FlpO* driver.

The second advance of the intersectional strategy is the high selectivity that can be achieved. Our intersectional breedings have routinely removed more than 90% of the cells in Cre drivers, and lead to handful cell types remaining in the intersection. When crossing *Pvalb-FlpE* with *Cck-Cre*, *Pcp2-Cre*, *Crh-Cre* or *Cart-Cre*, we were able to isolate only one to three types of RGCs, which represents a big improvement compared with most existing Cre lines (Zhu et al., 2014; Martersteck et al., 2017). A more striking example came from the *Penk-Cre*/*Sst-FlpO* intersection which removed most cell types in the *Penk-Cre* driver and narrowed cell types down to mainly just one type of AC in the INL and one type of RGC in the GCL. By selecting appropriate pairs of Cre and Flp drivers for crossing, an intersectional strategy holds out the possibility of labeling, or introducing opto- and chemo-genetic manipulations into only a single type.

A present-day weakness of our approach is that Flp drivers for targeting more specific cell types, in particular, the AC types, are still lacking, except for *Vip-FlpO* and *Sst-FlpO*. Future development of Flp drivers to target more specific ACs will greatly benefit the functional characterization of AC types, the least well characterized cell class in the retina. The prevalence of mouse lines that report the presence of two recombinases is also an issue. Several reporter mouse lines for delivering fluorescent markers and genetic tools are available, including Ai65 for tdTomato and Ai80 for calcium translocating channelrhodopsin (CatCh, JAX025109). Further development of reporter lines for other genetically encoded sensors and effectors, such as GCaMP, iGluSnFr and DREADDs, will greatly facilitate various ways of observing and manipulating functions of cell types in the Cre/Flp intersection.

Beyond enhancing the specificity for cell type targeting, intersectional strategies also provide a powerful platform for discovering new cell types. Identifying AC types and completing the anatomical catalog for this major class of retinal interneuron has been a long-term challenge. Judiciously chosen intersections restrict the number of targeted cell types and can greatly reduce the density of cell labeling. This reduction allows us to tease apart the intermingled neuron networks in the IPL making it easier to anatomically identify new cell types. By introducing reporter mouse lines (Madisen et al., 2015) and viral vectors (Fenno et al., 2014; Madisen et al., 2015; He et al., 2016), genetically encoded calcium indicators and optogenetic effectors can be delivered to these new cells for systematic functional analysis.

## Author Contributions

YZ, JX, and SHD designed the experiments. AJ, JX, SD, SC, and YZ performed the experiments and collected the data. YZ and SHD analyzed the data and interpreted the results. YZ, JX, and SHD wrote the manuscript draft. All the authors reviewed and approved the manuscript.

## Conflict of Interest Statement

The authors declare that the research was conducted in the absence of any commercial or financial relationships that could be construed as a potential conflict of interest.
